# The association between subchondral bone cysts and tibial cartilage volume and risk of joint replacement in people with knee osteoarthritis: a longitudinal study

**DOI:** 10.1186/ar2971

**Published:** 2010-03-31

**Authors:** Stephanie K Tanamas, Anita E Wluka, Jean-Pierre Pelletier, Johanne Martel-Pelletier, François Abram, Yuanyuan Wang, Flavia M Cicuttini

**Affiliations:** 1 Department of Epidemiology and Preventive Medicine, School of Public Health and Preventive Medicine, Monash University, Alfred Hospital, Commercial Rd, Melbourne 3004, Victoria, Australia; 2 Osteoarthritis Research Unit, University of Montreal Hospital Research Centre (CRCHUM), Notre-Dame Hospital, 1560 Rue Sherbrooke East, Montreal, Quebec H2L 4M1, Canada; 3 Arthro Vision Inc., 1560 Rue Sherbrooke East, Montreal, Quebec H2K 1B6, Canada

## Abstract

**Introduction:**

To examine the natural history of subchondral bone cysts and to determine whether knee cartilage loss and risk of joint replacement is higher in knees with cysts, compared with those with bone marrow lesions (BMLs) only or those with neither BMLs nor cysts.

**Methods:**

The symptomatic knee in 132 subjects with knee osteoarthritis (OA) was imaged by using magnetic resonance imaging at baseline and 2 years later. Tibial cartilage volume, subchondral bone cysts, and BMLs were measured by using validated methods. Knee arthroplasty over a 4-year period was ascertained.

**Results:**

Bone cysts were present in 47.7% of subjects, 98.1% of whom also had BMLs. Over a 2-year period, 23.9% of subjects had cysts progress, 13.0% developed new cysts, and 11.4% had cysts regress. Bone cysts at baseline were associated with lower medial and lateral tibial cartilage volume compared with those with BMLs only or those with neither (*P *for trend 0.004 and <0.001, respectively). Annual medial cartilage volume loss was greatest in those with bone cysts compared with those with BMLs only or those with neither (9.3%, 6.3%, and 2.6%, respectively; *P *for trend, <0.001). As the severity of bone abnormality in the medial compartment increased from no BMLs or cysts present, to BMLs only, to subchondral bone cysts present, the risk of knee replacement was increased (odds ratio, 1.99; 95% confidence interval (CI), 1.01 to 3.90; *P *= 0.05).

**Conclusions:**

When cysts are present, cartilage loss and risk of knee replacement are higher than if only BMLs are present, suggesting that cysts identify those most likely to benefit from prevention of disease progression. As cysts can regress, they may also provide therapeutic targets in knee OA.

## Introduction

Subchondral bone cyst formation is often encountered in osteoarthritis (OA) of the knee, particularly in advanced OA [1]. Visualised by using magnetic resonance imaging (MRI), subchondral bone cysts occur where the overlying cartilage has largely been eroded [2]. Two main theories are proposed about cyst formation: the synovial breach theory [3,4] and the bony contusion theory [1,5].

Subchondral bone cysts are present in ~50% of subjects with knee OA [6,7] and in 13.6% of healthy volunteers [8]. Studies of subchondral bone cysts have predominantly been descriptive, relating to the prevalence of subchondral bone cysts in OA [2,7,9,10]. Two recent studies that examined the relationship between subchondral bone cysts and knee pain found conflicting evidence [11,12]. A cross-sectional study of 143 subjects with knee OA reported no association between cysts and knee pain [12]. In contrast, a prospective study, which is part of an ongoing Genetics, Osteoarthritis, and Progression Study, of 205 subjects with knee OA found a trend for an association between subchondral bone cysts and increased risk of knee pain [11]. To our knowledge, the relationship between subchondral bone cysts and change in knee structure has been examined by only one study. This found a correlation between mean cyst size change (mm) and cartilage loss in the medial femoral condyle over a 24-month period [6]. No study has examined the presence of subchondral bone cysts at baseline as a risk factor for structural changes in the knee.

The relationship between bone marrow lesions (BMLs) and subchondral bone cysts is unclear, although it was recently proposed that BMLs may develop into subchondral bone cysts [13-15]. A small retrospective study of 32 patients with knee OA found that 11 (92%) of 12 of cysts developed within BMLs over ~18 months [13]. This is consistent with the findings of a more recent study of 400 patients with or at risk of knee OA, which showed that BMLs were coexistent in 91.2% of the subregions where cysts were found [14]. It may be that subchondral bone cysts indicate those with severe BMLs and more advanced disease.

In a population with symptomatic knee OA, this study aimed to (a) examine the natural history of subchondral bone cysts; and (b) determine whether tibial cartilage volume loss and risk of joint replacement is higher in knees with subchondral bone cysts, compared with those with bone marrow lesions (BMLs) only or those with neither BMLs nor cysts.

## Materials and methods

### Study population

Subjects with knee OA were recruited by advertising through local newspapers and the Victorian branch of the Arthritis Foundation of Australia and in collaboration with general practitioners, rheumatologists, and orthopedic surgeons. The study was approved by the ethics committee of the Alfred and Caulfield Hospitals in Melbourne, Australia. All subjects gave informed written consent [16].

One hundred thirty-two subjects entered the study. Inclusion criteria were age older than 40 years, knee symptoms (at least one pain dimension of Western Ontario and McMaster University Osteoarthritis Index (WOMAC [17]) score >20% and osteophytes present), and radiographic knee OA (ACR radiographic and clinical criteria [18]). Subjects were excluded if any other form of arthritis was present, MRI was contradicted (for example, pacemaker, cerebral aneurysm clip, cochlear implant, presence of shrapnel in strategic locations, metal in the eye, and claustrophobia), inability to walk 50 feet without the use of assistive devices, hemiparesis of either lower limb, or planned total knee replacement.

### Anthropometric and clinical data

Weight was measured to the nearest 0.1 kg (shoes and bulky clothing removed) by using a single pair of electronic scales. Height was measured to the nearest 0.1 cm (shoes removed) by using a stadiometer. Body mass index (BMI; weight/height^2 ^(kg/m^2^)) was calculated. Function and pain were assessed with WOMAC (VAS, 10 cm) [17].

### Radiograph

At baseline, each subject had a weight-bearing anteroposterior tibiofemoral radiograph of the symptomatic knee in full extension. Where both knees had OA and were symptomatic, the knee with least severe radiographic OA was used. These were independently scored by two trained observers who used a published atlas to classify disease in the tibiofemoral joint according to the Kellgren and Lawrence (K-L) scale. The radiologic features of tibiofemoral OA were graded in each compartment, on a 4-point scale (0 to 3) for individual features of osteophytes and joint space narrowing [19]. In the case of disagreement between observers, the films were reviewed by a third independent observer, and consensus values were used. Intraobserver reproducibility (κstatistic) for agreement on features of OA was 0.93 for osteophytes (grade 0, 1 versus 2, 3) and 0.93 for joint-space narrowing (grade 0, 1 versus 2, 3). Interobserver reproducibility was 0.86 for osteophytes and 0.85 for joint-space narrowing [20].

### Magnetic resonance imaging

Each subject had an MRI performed on the symptomatic knee at baseline and ~2 years later. Knees were imaged in the sagittal plane on the same 1.5-T whole-body magnetic resonance unit (Signa Advantage HiSpeed; GE Medical Systems, Milwaukee, WI) by using a commercial receive-only extremity coil. The following sequence and parameters were used: a T_1_-weighted fat-suppressed 3D gradient recall acquisition in the steady state; flip angle, 55 degrees; repetition time, 58 msec; echo time, 12 msec; field of view, 16 cm; 60 partitions; 512 × 192 matrix; one acquisition time, 11 min 56 sec. Sagittal images were obtained at a partition thickness of 1.5 mm and an in-plane resolution of 0.31 × 0.83 mm (512 × 192 pixels).

Knee cartilage volume was determined by means of image processing on an independent work station by using the software program Osiris, as previously described [16,20]. Two trained observers read each MRI. Each subject's baseline and follow-up MRI scans were scored unpaired and blinded to subject identification and timing of MRI. Their results were compared. If the results were within ± 20%, an average of the results was used. If they were outside this range, the measurements were repeated until the independent measures were within ± 20%, and the averages were used [16,20]. Repeated measurements were made blind to the results of the comparison of the previous results. The coefficients of variation (CVs) for the measurements were 3.4% for the medial, 2.0% for the lateral, and 2.6% for the total tibial cartilage volume [16]. Tibial plateau area was determined by creating an isotropic volume from the three input images closest to the knee joint, which were reformatted in the axial plane. The area was directly measured from these images. The CVs for the medial and lateral tibial plateau area were 2.3% and 2.4%, respectively [16,20].

A subchondral bone cyst was defined as a well-demarcated hypersignal, whereas a BML was an ill-defined hypersignal. The assessments of subchondral bone cysts and BMLs were performed on the MRI slice that yielded the greatest lesion size. The intensity and extent of cysts and BMLs were assessed in the medial and lateral tibiofemoral compartments and were graded as 0, absence of lesion; 1, mild to moderate lesion; and 2, severe (large) lesion. A reliability study done by using a two-reader consensus measure of a specific lesion size twice at a 6-week interval showed an *r *= 0.96, *p *< 0.0001 for subchondral bone cysts and *r *= 0.80, *p *< 0.001 for BMLs (test-retest Spearman correlation) [6]. The medial and lateral cyst and BML scores were each calculated as a sum of the scores for the tibial, femoral, and femoral posterior sites (scores 0 to 6). As a low prevalence of subjects was found with cyst scores >3 for the medial and >1 for the lateral compartment, we collapsed the scores to give a range of 0 to 3 for the medial and 0 to 1 for the lateral compartment.

### Identification of knee replacement

At year 4, all subjects were contacted and asked whether they had undergone a knee replacement because of OA of the same knee in which they had a baseline MRI. This was confirmed by contacting the treating physician in all cases.

### Statistical analysis

Descriptive statistics for characteristics of the subjects were tabulated. Annual percentage change in cartilage volume was calculated by cartilage change (follow-up cartilage volume subtracted from initial cartilage volume) divided by initial cartilage volume and time between MRIs. Outcome variables (baseline tibial cartilage volume and annual percentage change in tibial cartilage volume) were initially assessed for normality and were found to approximate normal distribution. Estimated marginal means was used to explore the cross-sectional relationship between subchondral bone cysts and tibial cartilage volume at baseline, and longitudinally, the relationship between baseline subchondral bone cysts and annual percentage tibial cartilage volume loss. Logistic regression was used to examine the relationship between baseline subchondral bone cysts and risk of knee-joint replacement over a 4-year period. All analyses were performed by using the SPSS statistical package (version 16.0.0; SPSS, Cary, NC), with a *P *value < 0.05 considered statistically significant.

## Results

Of the 132 subjects who took part in our study, 23 did not have an MRI from which subchondral bone cysts could be assessed (MRI not available or image unclear). The 109 subjects analyzed had a mean age of 63.2 (SD ± 10.3) years, and a mean BMI of 29.3 (SD ± 5.1) kg/m^2^. Demographics were not different between those who were included in the study and those who were not (data not shown). Eighty-eight (81%) subjects completed the follow-up; 21 were lost to follow-up for reasons including knee surgery, severe illness, loss of interest, death, and unclear MRI images from which cysts could not be assessed. Those who completed the follow-up had a lower mean BMI than did those who did not (mean ± SD, 28.8 ± 5.0 and 31.3 ± 5.4, respectively; *P *= 0.05).

Fifty-two (47.7%) subjects had at least one subchondral bone cyst at baseline. They were more likely to be male subjects, although no significant difference was found in age, weight, height, or BMI. Those with cysts had less lateral tibial cartilage volume and greater tibial plateau bone area compared with those who did not have a cyst (Table 1). Of subjects with a cyst at baseline, 98.0% also had a BML (Table 1). Furthermore, those with subchondral bone cysts were more likely to have large BMLs (grade ≥ 3). In contrast, those with a BML but no cyst at baseline tended to have small BMLs (grade 1).

**Table 1:** Comparison of characteristics between subjects

	Cyst present (n = 52)	No cyst (n = 57)	*P *value
Age (years)	64.5 (10.3)	62.1 (10.1)	0.22^a^
Female, number (%)	21 (40.4)	35 (61.4)	0.03^b^
Height (cm)	168.9 (9.6)	167.8 (8.4)	0.55^a^
Weight (kg)	83.1 (15.6)	83.0 (15.2)	0.98^a^
Body mass index (kg/m^2^)	29.1 (4.9)	29.5 (5.5)	0.67^a^
Kellgren-Lawrence grade ≥ 2, number (%)	37 (72.5)	41 (77.4	0.57^b^
Medial tibial cartilage volume (mm^3^)	1,819 (511)	1,769 (454)	0.58^a^
Lateral tibial cartilage volume (mm^3^)	1,855 (619)	2,156 (522)	0.01^a^
Medial tibial bone area (mm^2^)	2,246 (405)	1,976 (349)	<0.001^a^
Lateral tibial bone area (mm^2^)	1,446 (243)	1,292 (229)	0.001^a^
Tibiofemoral BML present, number (%)	51 (98.1)	21 (36.8)	<0.001^b^
Knee-joint replacement over 4 years, number (%)	9 (19.6)	7 (13.7)	0.44^b^

BML, bone marrow lesion. Presented as mean (SD), unless otherwise stated. *P *value calculated by using independent sample *t *test^a ^or χ^2 ^test^b^.

Twenty-one (23.9%) subjects had a cyst that increased in score over a 2-year period (cyst progression), including 6 (13.0%) in whom one or more subchondral bone cysts developed (Table 2). All had a coexisting BML at baseline. Of those with a cyst at baseline, cyst progression was observed in 15 (35.7%) subjects, whereas a decrease in cyst score (cyst regression) was observed in 10 (23.8%) subjects, with 6 (14.3%) resolving completely (Table 2). No change in cyst (stable) was observed in the remaining 17 (40.5%) subjects.

**Table 2:** Natural history of subchondral bone cysts

	Whole population (n = 109)	No BML or cyst at baseline (n = 36)	BML at baseline (n= 21)	Cyst at baseline (n = 52)
	No. (%)^a^	No. (%)^b^	No. (%)^c^	No. (%)^d^
Develop	6 (6.8)	0	6 (40.0)	N/A
Progress	21 (23.9)	N/A	N/A	15 (35.7)
Regress	10 (11.4)	N/A	N/A	10 (23.8)
Resolve	6 (6.8)	N/A	N/A	6 (14.3)
Stable	17 (19.3)	N/A	N/A	17 (40.5)

BML, bone marrow lesion; N/A, not applicable. ^a^88 subjects; ^b^31 subjects; ^c^15 subjects; and ^d^42 subjects of each subgroup participated in the follow-up; thus, the percentages were calculated accordingly.

The mean cartilage volume was lower in both compartments in those with cysts, compared with those with BMLs only or neither cyst nor BML present (Table 3). In the medial compartment, those with cysts present had a mean medial cartilage volume of 1,589 mm^3 ^compared with a mean of 1,809 mm^3 ^in those with BMLs only and 1,923 mm^3 ^in those with neither (*P *for trend, 0.004). Similarly those with cysts also had the least amount of lateral tibial cartilage volume compared with those with BMLs only or neither (mean, 1,607, 1,962, and 2,131 mm^3^, respectively; *P *for trend, <0.001). In the longitudinal analyses (Table 3), those with cysts had the highest rate of cartilage loss (9.3%) compared with the other two groups (6.3% and 2.6%) (*P *for trend, <0.001). Similar results were obtained when the subject with a cyst but no BML was excluded.

**Table 3:** Relation between increasing grade of severity of subchondral bone abnormality and tibial cartilage volume

	No BML or cyst at baseline Mean (95% CI)	With BML at baseline Mean (95% CI)	With cyst at baseline Mean (95% CI)	*P *for trend
Medial tibial cartilage volume^a^	1,923 (1,808, 2,038)	1,809 (1,640, 1,979)	1,589 (1,442, 1,735)	0.004
Lateral tibial cartilage volume^b^	2,132 (2,028, 2,236)	1,962 (1,616, 2,309)	1,607 (1,399, 1,817)	<0.001
Medial tibial cartilage volume loss^a^	2.62 (0.82, 4.42)	6.30 (3.43, 9.17)	9.26 (6.78, 11.73)	<0.001
Lateral tibial cartilage volume loss^b^	5.88 (4.18, 7.59)	7.19 (1.46, 12.93)	2.42 (-1.00, 5.84)	0.17

Volume expressed as cubic millimeters. Abnormality: 1, normal; 2, BML only; 3, both BML and cyst present. ^a^Association with cysts and BMLs in the medial compartment. ^b^Association with cysts and BMLs in the lateral compartment. Mean, 95% confidence interval, and *P *value were calculated by using Estimated Marginal Means. CI, confidence interval; BML, bone marrow lesion.

We extended our observation by examining the effect of increasing grade of severity of subchondral bone abnormality (grade 1, normal; 2, BMLs only; 3, BML and cyst present) on risk of knee-joint replacement over a 4-year period (Table 4). For every one grade increase in severity of bone abnormality in the medial compartment, the risk of joint replacement was increased (odds ratio, 1.99; 95% CI, 1.01 to 3.90; *P *= 0.05) when adjusted for age, gender, and K-L grade. No significant association was found in the lateral compartment. Again, similar results were obtained when excluding the subject with a cyst but no BML.

**Table 4:** Effect of increasing grade of severity of subchondral bone abnormality on joint replacement

	Univariate analysis OR (95% CI)	*P *value	Multivariate analysis^a^ OR (95% CI)	*P *value
Medial TF compartment	1.72 (0.93 to 3.18)	0.08	1.99 (1.01 to 3.90)	0.05
Lateral TF compartment	0.95 (0.48 to 1.88)	0.89	0.96 (0.48 to 1.94)	0.91

Abnormality: 1, normal; 2, BML only; 3, both BML and cyst present. ^a^Adjusted for age, gender, and Kellgren-Lawrence grade. OR, odds ratio; CI, confidence interval; TF, tibiofemoral.

When we examined the effect of change in subchondral bone cyst on cartilage, we found that those who had cyst regression in the lateral compartment had significant reduction in lateral tibial cartilage loss (regression coefficient, -11.81; 95% CI, -16.64 to -6.98; *P *< 0.001) compared with those who were stable or progressed. However, those who had cyst progression tended to have greater medial cartilage loss (regression coefficient, 3.51; 95% CI, -0.35 to 7.37; *P *= 0.07) than did those who were stable or regressed, although the results did not reach significance. Sixteen (33.3%) subjects had a knee-joint replacement over a 4-year period (Table 1). Because of the low numbers of progression and regression (one and three subjects, respectively) in this group, we could not examine the relationship between cyst change and risk of joint replacement.

## Discussion

In a population with symptomatic knee OA, subchondral bone cysts were common and usually coexisted with BMLs. They showed a varied natural history over a 2-year period, including the development of new cysts and the progression of existing cysts, as well as regression in size, including occurrence of complete resolution. Subjects with cysts had lower mean tibial cartilage volume at baseline, and greater loss of medial tibial cartilage volume over a 2-year period in longitudinal analyses, as well as an increased risk of knee-joint replacement over a 4-year period. Our findings suggest that having a subchondral bone cyst is associated with more severe structural changes and worse clinical outcomes compared with knees having BMLs only or having neither.

**Figure 1:**
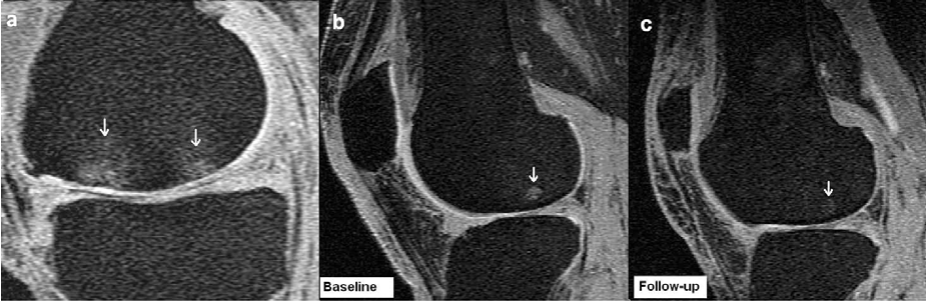
**(a) Grade 2 medial femoral bone marrow lesions**. **(b) **Lateral femoral subchondral bone cyst at baseline. **(c) **Regression of lateral femoral subchondral bone cyst at follow-up.

Subchondral bone cysts were present in 48% of our study population, similar to the prevalence reported in previous studies [6,7]. As observed in other studies, cysts were found to coexist commonly with BMLs [13-15], particularly large BMLs of grade 3 or higher. Few studies have examined the natural history of subchondral bone cysts. In a randomized double-blind placebo controlled trial of risedronate treatment in 107 subjects with knee OA, although no effect of risedronate therapy was observed on bone lesions (BMLs and cysts), the average size of subchondral bone cysts increased over a 24-month period [6]. However, this study [6] looked only at mean cyst-size change over a 24-month period without discrimination between regression and progression. In the present study, we found that although it was most common for cysts to increase in size, a significant proportion regressed (Figure 1), including complete resolution.

When we examined subchondral bone cysts in relation to knee structure, we found that having a cyst was associated with reduced cartilage volume, increased cartilage loss, and increased risk of knee replacement compared with having BMLs only or having neither. No previous study has examined the effect of cysts and BMLs separately. One previous study found that increased size of subchondral bone cysts (both with and without BMLs) was correlated with cartilage loss in the medial femoral condyle [6]; however, the association between the presence of cysts at baseline and cartilage volume was not examined. We also found that those who had an increase in cyst score tended to lose more medial tibial cartilage, whereas regression of cysts was associated with reduced loss of lateral tibial cartilage. It may be that some of the compartment differences observed are due to the modest sample size. However, taken together, these results suggest that subchondral bone cysts identify those likely to have adverse structural outcomes and that regression of cysts is protective against cartilage loss.

**Figure 2:**
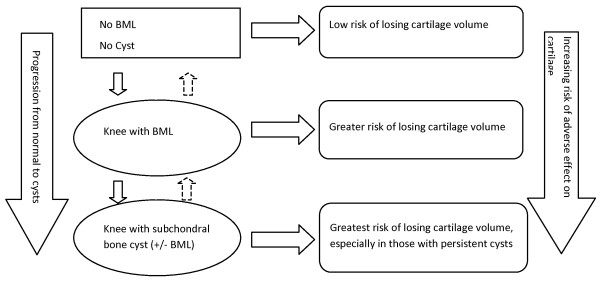
**The progression from normal to subchondral bone cysts and its relation with cartilage**.

Subchondral bone cysts were initially thought to result from degenerative changes to cartilage, creating a communication between subchondral bone and the synovial space, allowing breach of synovial fluid into the marrow space [4,5]. However, subsequent evidence supports the bony contusion theory, in which violent impact between opposing surfaces of the joint results in areas of bone necrosis, particularly when the overlying cartilage has been eroded, and that synovial breach is a secondary event [1,5,14]. Recent studies have shown that cysts may develop in preexisting BMLs, leading to the proposed theory that BMLs may in fact be early "pre-cystic" lesions [13,15]. The results of our study support this notion. However, given that BMLs are the result of a number of different pathogenetic mechanisms, which include both traumatic and nontraumatic mechanisms, it may be that cysts do not develop in all BMLs, but rather in some subgroups, and represent later stages of the pathologic process (Figure 2). Our data suggest that cysts identify those who tend to have worse knee outcomes and who should be particularly targeted for prevention of disease progression.

Several limitations to our study exist. Because of the moderate sample size of the current study, cyst progression was defined simply as an increase in score, and thus included both those who had an increase in score and incident cysts. Similarly, cyst regression was defined as a decrease in score, which did not differentiate those that resolved completely. A larger sample or a longer follow-up period or both will be required to examine further the relationship between subchondral cyst changes and knee structure. Additionally, because T_2_-weighted MRI was not available when we started our study, we used T_1_-weighted MRI to measure BMLs, which is likely to result in a more-conservative analysis. For BMLs to be identified on T_1 _images, BMLs must be larger and more active with surrounding edema [21,22]; thus, any BMLs identified on T_1 _images are likely to be definite and larger than were the T_2 _images used.

## Conclusions

In this study, we found that subchondral bone cysts tend to coexist with BMLs. When cysts are present, they identify patients with worse structural knee outcomes, including increased cartilage loss and increased risk of knee-joint replacement, than patients with BMLs only, and who may most benefit from prevention of disease progression. As we show that not only can cysts regress, but that regression also is associated with reduced cartilage loss, cysts may provide therapeutic targets in the treatment of knee OA.

## Abbreviations

BMI: body mass index; BML: bone marrow lesion; CI: confidence interval; CVs: coefficients of variation; MRI: magnetic resonance imaging; OA: osteoarthritis; OR: odds ratio; SD: standard deviation; WOMAC: Western Ontario and McMaster University Osteoarthritis Index.

## Competing interests

The authors declare that they have no competing interests.

## Authors' contributions

SKT was involved in data analyses and manuscript preparation. AEW was involved in manuscript preparation. JPP, JMP, and FA were involved in data collection and manuscript revision. YW was involved in data collection and manuscript revision. FMC was involved in manuscript preparation.
